# Structural and Functional Basis for p38-MK2-Activated Rsk Signaling in Toll-Like Receptor-Stimulated Dendritic Cells

**DOI:** 10.1128/MCB.00773-14

**Published:** 2014-12-09

**Authors:** Rossana Zaru, Alexander J. Edgar, André Hanauer, Colin Watts

**Affiliations:** a Division of Cell Signalling and Immunology, College of Life Sciences, University of Dundee, Dundee, United Kingdom; b Institute of Genetics and Molecular and Cellular Biology (IGBMC), Centre National de la Recherche Scientifique (UMR 7104), Institut National de la Santé et de la Recherche Médicale (U 964), University of Strasbourg, Illkirch, France

## Abstract

Rsk kinases play important roles in several cellular processes such as proliferation, metabolism, and migration. Until recently, Rsk activation was thought to be exclusively initiated by Erk1/2, but in dendritic cells (DC) Rsk is also activated by p38 mitogen-activated protein (MAP) kinase via its downstream substrates, MK2/3. How and why this noncanonical configuration of the MAP kinase pathway is adopted by these key immune cells are not known. We demonstrate that the Erk1/2-activated C-terminal kinase domain of Rsk is dispensable for p38-MK2/3 activation and show that compared with fibroblasts, a greater fraction of p38 and MK2/3 is located in the cytosol of DC prior to stimulation, suggesting a partial explanation for the operation of the noncanonical pathway of Rsk activation in these cells. p38/MK2/3-activated Rsk phosphorylated downstream targets and is physiologically important because in plasmacytoid DC (pDC) stimulated with Toll-like receptor 7 (TLR7) agonists, Erk1/2 activation is very weak relative to p38. As a result, Rsk activation is entirely p38 dependent. We show that this unusual configuration of MAP kinase signaling contributes substantially to production of type I interferons, a hallmark of pDC activation.

## INTRODUCTION

Different cells share many signaling pathway components yet are able to propagate cell-specific signaling events leading to distinct outcomes. How this is achieved is incompletely understood. In most cells, a key pathway is the Mek1/2-Erk1/2 mitogen-activated protein (MAP) kinase pathway leading to downstream activation of many key substrates, including the MAP kinase-activated kinases Msk1/2 and Rsk1 to Rsk3 (reviewed in reference 1). A striking feature of these closely related kinases is the presence of distinct N- and C-terminal kinase domains (NTKD and CTKD) with the latter playing a key role in activation of the former. The seminal studies of this pathway showed that while Msk1/2 can be activated by both Erk1/2 and p38 MAP kinases (2), Rsk kinases are exclusively activated by Erk1/2 (2). In earlier work, we demonstrated an important exception to this rule in Toll-like receptor (TLR)-stimulated murine dendritic cells (DC) by showing that Rsk is also activated downstream of p38 signaling via the intermediate kinases MK2/3 (3). The two pathways for Rsk activation converged at Ser380/386 in Rsk1 and -2, respectively, a key site lying in a “hydrophobic motif” between the two kinase domains that is responsible for recruitment of the NTKD-activating kinase PDK1 (4, 5). In the canonical pathway, this key site is phosphorylated in *cis* by the Rsk C-terminal kinase domain (CTKD) (6, 7), whereas in the alternative pathway, the site is phosphorylated in *trans* by MK2/3, apparently bypassing the CTKD (3). A CTKD-independent pathway for Rsk activation was also demonstrated in lipopolysaccharide (LPS)-stimulated macrophages (8).

Dendritic cells are crucial for the initiation of immune responses. They are able to sense pathogens and mount an appropriate immune response tailored to the pathogens encountered (9). Due to their capacity to process and present pathogen-derived antigens and produce pro- and anti-inflammatory cytokines, they activate T lymphocytes. Several DC subsets such as plasmacytoid DC (pDC) and conventional CD8^+^ and CD8^−^ DC have been described (10). Although these different DC populations share some similar features, they also have specific functions. For instance, pDC respond to viral infection by producing large amounts of type I interferons (IFN). DC sense pathogens through several receptors such as those in the Toll-like receptor family that recognize pathogen-derived products such as lipopolysaccharide (LPS) (11, 12). The signaling cascades activated following TLR engagement include the p38 and Erk1/2 pathways (13).

TLR expression is not limited to cells of the innate immune system such as DC or macrophages but is also found in T and B lymphocytes (14, 15) and indeed in nonimmune cells such as fibroblasts and endothelial and epithelial cells (12). Several studies have shown that although these different cell types recognize the same TLR ligand, their responses are unique. For instance, in B cells, TLR9 stimulation induced not only the production of cytokines, as in DC or macrophages but also their proliferation (16). Apparently, the wiring of TLR signaling cascades is different in different cell types.

In this study, we investigated several important questions raised by our earlier finding of a p38-MK2/3-driven pathway of Rsk activation in DC. For example, what are the structural requirements for activation of Rsk when the CTKD is apparently bypassed? Does the C-terminal region of Rsk still play a role? Do Erk1/2-activated Rsk and p38-activated Rsk have the same downstream substrates? Also, since p38-MK2 signaling is intact in the many cell types that activate Rsk exclusively via Erk1/2, what prevents the p38-MK2 signaling module from activating Rsk in these cells? Perhaps most important, what is the functional significance of p38-MK2-mediated Rsk activation? By comparing DC with other immune and nonimmune cells, we provide some answers to these questions.

## MATERIALS AND METHODS

### Mice and cell culture.

Rsk2^−/−^ mice have been described previously in reference 17. Bone marrow-derived dendritic cells (BMDC) were expanded from C57BL/6 or Rsk2^−/−^ bone marrow using complete RPMI 1640 medium (cRPMI) supplemented with 10 ng/ml recombinant granulocyte-macrophage colony-stimulating factor (GM-CSF) (Peprotech) as described in reference 18. Murine embryonic fibroblasts (MEF) were generated from C57BL/6 or Rsk2^−/−^ mice and maintained as described previously (3). Bone marrow-derived macrophages (BMMϕ) were expanded from bone marrow using complete Dulbecco modified Eagle medium (DMEM) supplemented with 30% L929 supernatant for 7 to 9 days. B cells were isolated by negative selection from the spleen. Briefly, spleen cells were incubated 30 min on ice with biotin-coupled anti-CD4, anti-CD8, anti-CD11c, and anti-Ly6G antibodies (all from eBioscience). The cells were washed and incubated with streptavidin Dynal beads (Invitrogen) for 20 min at room temperature. The cells were 91% positive when stained with allophycocyanin (APC)-labeled anti-CD19 (eBioscience). pDC were generated from bone marrow in the presence of 100 ng/ml Flt3 ligand (Flt3-L) (Peprotech) in cRPMI for 10 days. pDC (B200^+^) were sorted on an Influx cell sorter (BD Biosciences). Cells were 98% positive when stained with fluorescein isothiocyanate (FITC)-labeled anti-B220 antibodies (eBioscience). Animal work was overseen by a local Ethical Review Committee and was conducted in accordance with United Kingdom Home Office Project Licenses.

### Retroviral constructs and retroviral infection of BMDC and MEF.

Moloney murine leukemia virus (MoMLV)-based pBMN-I-GFP (GFP stands for green fluorescent protein) retroviral vector was provided by G. Nolan, Stanford. Rsk2 was cloned from BMDC cDNA. A myc tag was inserted at the N terminus by PCR (5′-ATGGAGCAGAAGCTGATCAGCGAGGAGGACCTGCCGCTG GCGCAGCTGGC-3′). Full-length Rsk2 and truncated forms of Rsk2 were generated by PCR with the following primers for the C-terminal region: 5′-TCACAGGGCTGTTGAGGTG-3′ for the full-length, myc-tagged Rsk2 (amino acids 1 to 740) [myc Rsk2 (1–740)], 5′-AAATGCGGCCGCACTCCGCTGAGCAAGAGTGGAGCG-3′ for myc Rsk2 (1–728), or 5′-TCAAATAGCAACAAAACTAAACCCCC-3′ for myc Rsk2 (1–390). The full-length and truncated forms of Rsk2 were cloned into the BamHI and NotI sites of pBMN-I-GFP. Virus was produced by transfecting the Phoenix Eco 293T packaging cell line as previously described (18). MEF were plated at 8 × 10^4^ in 6-cm dishes. After 24 h, cells were infected with 1 ml of virus supplemented with 5 μg/ml Polybrene (Sigma). BMDC were infected at day 2 and day 3 with viral supernatant supplemented with 8 μg/ml Polybrene as described in reference 18.

### Cell stimulation and cell lysate preparation.

A total of 10^6^ BMDC or BMMϕ were incubated for 5 h at 37°C in RPMI containing 0.3% fetal calf serum (FCS) in 6-well plates. Cells left untreated or treated with either dimethyl sulfoxide (DMSO) or 2 μM PD184352 (provided by the Division of Signal Transduction Therapy [DSTT], University of Dundee), 0.1 μM BIRB796 (DSTT), 4 μM BI-D1870 (kind gift of Boehringer-Ingelheim Pharmaceuticals) for 30 min, or 2 μM BIX02565 (kind gift of Boehringer-Ingelheim Pharmaceuticals) for 1 h at 37°C, or 50 nM leptomycin B (Merck Millipore) for 15 min at 37°C were stimulated for 30 min with either 50 ng/ml LPS (Axxora) or 1 μM CpG B (Invivogen) for 30 min or with 1 μg/ml R848 (Axxora) for 15 min at 37°C. MEF were starved overnight at 37°C, pretreated with inhibitors as described above, and then stimulated with 200 ng/ml LPS for 30 min at 37°C. Purified B cells (2 × 10^6^ cells) were incubated in RPMI supplemented with 5% FCS for 1 h in 48-well plates. Inhibitors were added as described above followed by the addition of 1 μM CpG B for 30 min at 37°C. Sorted pDC (10^5^ cells) were incubated in 1.5-ml conical tubes for 1 h at 37°C in RPMI containing 2% FCS. Inhibitors were added as described above followed by the addition of 1 μg/ml R848 or 0.1 μM CpG B for different lengths of time at 37°C.

Cells were washed once in cold phosphate-buffered saline (PBS) and lysed in 1% Triton X-100 containing 50 mM Tris-HCl (pH 7.5), 1 mM EGTA, 1 mM EDTA, 1 mM sodium orthovanadate, 10 mM sodium fluoride, 5 mM sodium pyrophosphate, and 0.1% β-mercaptoethanol and 1 tablet of protease inhibitors (Roche) for 10 min on ice. Lysates were centrifuged for 10 min at 20,000 × *g* at 4°C. Equal amounts of proteins were loaded on 4 to 12% NuPage gels (Invitrogen) and then transferred onto nitrocellulose membranes (Amersham). Membranes were probed with the following antibodies. Antibodies to phosphorylated Rsk (p-Rsk) (S386) and p-Rsk (S227) were from R&D Systems. Antibodies to p-Rsk (T573) (clone 9346), Rsk2 (clone 9340), p-Erk1/2 (clone 9101), p-p38 (clone 9211), phosphorylated glycogen synthase kinase 3α/β (p-GSK3α/β) (S21/S9) (clone 9331), GSK3β (clone 9315), phosphorylated filamin A (S2152) (clone 4761), p-MK2 (clone 2111), and MK2 (clone 3042) were from Cell Signaling. Antibodies to the myc tag (9E10; Millipore), p-NHE1 (S703) (DSTT), NHE1 (catalog no. 611774; BD Bioscience), and filamin A (H-300; Santa Cruz Biotechnology) were from the indicated companies. In some experiments, lysates were precleared with protein G-Sepharose (Amersham) and incubated with 2 μg of either anti-RSK2, anti-NHE1, or anti-myc antibodies for 1 h at 4°C followed by the addition of 15 μl of protein G-Sepharose for 1 h at 4°C.

### Microscopy.

2 × 10^5^ BMDC or 50 × 10^4^ MEF were plated on glass coverslips in RPMI supplemented with 0.3% FCS or DMEM supplemented with 2 mg/ml bovine serum albumin (BSA) for 2 h or overnight, respectively. The cells were fixed in 4% paraformaldehyde in PBS for 15 min and permeabilized with 100% ice-cold methanol (MeOH) for 10 min on ice. After the cells were blocked with 1% BSA in Tris-buffered saline (TBS), they were stained with either anti-p38, anti-MK2 or anti-MK3 antibodies (all from Cell Signaling) followed by Alexa Fluor 488-conjugated donkey anti-rabbit antibodies and 4′,6′-diamidino-2-phenylindole (DAPI) (Invitrogen). Single optical sections were acquired on a confocal microscope (LSM 700) using a 40× or 100× lens objective (Carl Zeiss, Inc.).

### Rsk kinase assay.

BMDC were stimulated as described above, and lysates were prepared as described above. The supernatant was frozen in liquid nitrogen and stored at −80°C. Endogenous Rsk2 was immunoprecipitated with 2 μg of anti-Rsk2 antibodies (Santa Cruz Biotechnology). Wild-type Rsk2 and mutant forms of Rsk2 were immune precipitated from 25 μg of lysates with 2 μg of anti-myc coupled to protein G-Sepharose for 30 min at 4°C. After 4 washes with buffer A (50 mM Tris-HCl [pH 7.5], 0.1 mM EGTA, and 0.1% β-mercaptoethanol), the beads were resuspended in 50 mM Tris-HCl (pH 7.5), 0.1 mM EGTA, 0.1% β-mercaptoethanol, 2.5 μM protein kinase inhibitor (PKI) (TTYADFIASGRTGRRNAIHD; peptide inhibitor of cyclic-AMP-dependent protein kinase), 10 mM magnesium acetate, 0.1 mM [γ-^32^P]ATP, and Crosstide (GRPRTSSFAEG; 30 μM). The kinase assay was carried out for 30 min at 30°C and then terminated and analyzed as described previously (19).

### Cytokine production.

pDC (7 × 10^4^ cells) were incubated for 15 min in 96-well round bottom plates in complete RPMI. Cells were either untreated or pretreated with 2 μM PD184352 or with 0.1 μM BIRB796 for 30 min or with 2 μM BIX02565 for 1 h at 37°C before being stimulated with 1 μg/ml R848 (Axxora) for 18 h. The amount of interferon beta (IFN-β) was measured by enzyme-linked immunosorbent assay (ELISA) (IFN-β; R&D Systems).

## RESULTS

### Activation of Rsk by the p38 pathway is limited to some hematopoietic cells.

**FIG 1:**
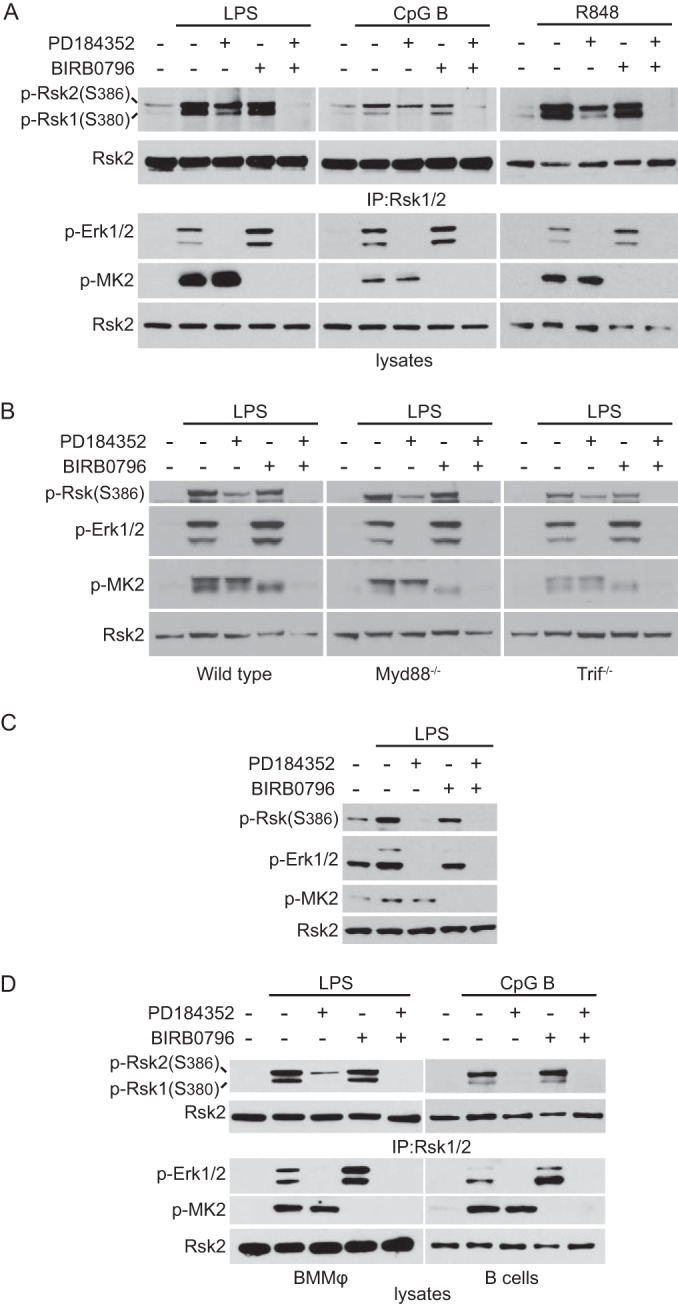
Rsk activation by the p38 pathway is restricted to BMDC and BMMϕ. Immunoblot analysis of the phosphorylation of Rsk1/2 at Ser380/386, Erk1/2, and MK2. (A and B) Wild-type (A and B), Myd88^−/−^ (B), or Trif^−/−^ (B) BMDC were left untreated (−) or stimulated (+) with LPS (50 ng/ml) or CpG B (1 μM) for 30 min or R848 for 15 min (leftmost portion of panel A). The lysates were immunoprecipitated (IP) with Rsk1/2 antibodies. (C) MEF were left untreated or stimulated with LPS (200 ng/ml) for 30 min. (D) BMMϕ or B cells were left untreated or stimulated with LPS (50 ng/ml) or CpG B (1 μM) for 30 min. (A to D) Cells were stimulated in the presence (+) or absence (−) of 2 μM PD184352 or 0.1 μM BIRB0796. The results shown are representative of the results from at least three independent experiments.

We first confirmed and extended our earlier finding that distinct pathways are used to activate Rsk in DC compared with other cell types such as MEF. Activation of Rsk requires phosphorylation of a key serine residue in a hydrophobic motif (Ser380 and Ser386 in Rsk1 and Rsk2, respectively) which lies between the two kinase domains and recruits the NTKD-activating kinase PDK1. As shown in Fig. 1A, stimulation of bone marrow-derived DC (BMDC) with ligands for TLR4 (LPS), TLR9 (CpG), and TLR7 (R848) resulted in robust phosphorylation of this site. However, in DC, Ser380/386 phosphorylation was resistant to inhibition of the Erk1/2 activating kinases MEK1/2 by PD184352 and could be fully blocked only when the p38 MAP kinase inhibitor BIRB0796 was also included (Fig. 1A). Control experiments showed that both MAP kinase pathways were fully inactivated by their respective inhibitors, because phosphorylation of Erk1/2 was completely blocked by PD184352 and phosphorylation of the p38 substrate MK2 was fully inhibited by BIRB0796 (Fig. 1A). Interestingly, the noncanonical p38 pathway of Rsk activation appeared more dominant for Rsk2 than for Rsk1 (Fig. 1A). Sustained activation of Erk1/2 and p38 via TLR4 signaling depends on sequential involvement of 2 TLR adaptors, MyD88 and TRIF (20, 21). To assess whether p38-mediated activation of RSK utilized one pathway in preference to the other, we stimulated DC lacking MyD88 or TRIF. As shown in Fig. 1B, both adaptor configurations of TLR4 were able to activate Rsk in a p38-dependent, Erk1/2-independent fashion. These results contrasted with the situation in most other cell types, including MEF where Rsk activation, even by the same TLR stimulus, was due to Erk1/2 rather than p38 signaling (Fig. 1C). Taken together with our earlier data (3), these results show that noncanonical Rsk activation by p38/MK2/3 following TLR signaling, is cell type specific rather than TLR stimulus specific.

As DC and fibroblasts have distinct origins and functions, we next asked whether p38-driven Rsk activation was possible in other cells of the hematopoietic lineage. We first looked in bone marrow-derived macrophages (BMMϕ), since they have the same myeloid origin as DC. As in DC, Rsk activation was still observed, albeit at a reduced level, when Erk1/2 signaling was blocked in BMMϕ and eliminated only when the p38 pathway was also suppressed (Fig. 1D, left). Again, this was most clearly seen in the case of Rsk2 (Fig. 1D). This is consistent with earlier results in LPS-stimulated BMMϕ showing persistent S386 phosphorylation in the presence of an inhibitor of the Rsk CTKD (8). Next we looked at B cells directly isolated from the spleen. Conversely to BMDC and BMMϕ, the contribution of the p38 pathway to Rsk activation in B cells was barely detectable (Fig. 1D, right). These results demonstrate that while the Erk1/2 pathway of Rsk activation operates in all cells, the capacity of p38 to activate Rsk is limited to certain hematopoietic cells, notably DC and macrophages.

### Structural requirements for p38-MK2/3 activation of Rsk.

**FIG 2:**
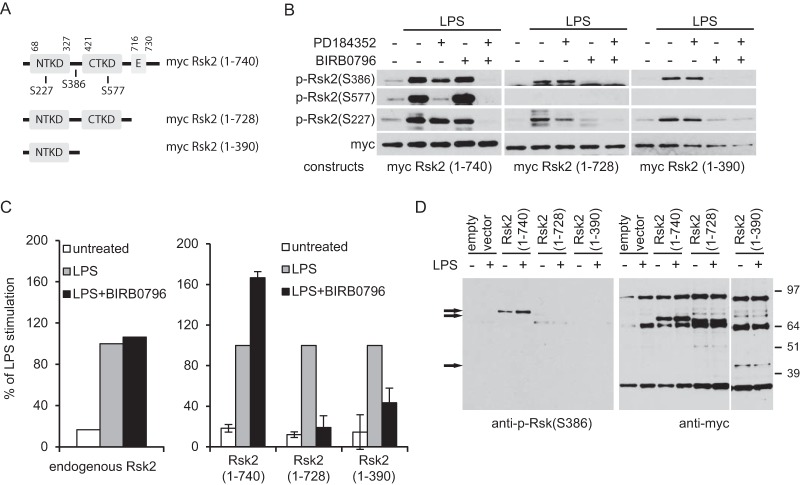
CTKD and Erk1/2 binding domain are dispensable for Rsk activation by the p38 pathway. (A) Wild type and truncated forms of myc Rsk2. NTKD, N-terminal kinase domain; CTKD, C-terminal kinase domain; E, Erk1/2 binding domain. (B) Rsk2^−/−^ BMDC were reconstituted with the indicated myc Rsk2 constructs. Cells were left untreated or were stimulated with LPS (50 ng/ml) for 30 min in the presence or absence of 2 μM PD184352 or 0.1 μM BIRB0796. Immunoblots were analyzed for Rsk phosphorylation at Ser577, Ser386, and Ser227. The results shown are representative of the results of three independent experiments. (C) Endogenous Rsk2 (left) and myc Rsk2 constructs (right) activity in BMDC left untreated or treated with LPS (50 ng/ml) for 30 min in the presence or absence of 0.1 μM BIRB0796. Data are the means ± standard deviations (SD) (error bars) of duplicate experiments and are representative of the results from two independent experiments. (D) Rsk2^−/−^ MEF infected with the indicated myc Rsk2 constructs were left untreated or stimulated with LPS (200 ng/ml) for 30 min. Rsk2 phosphorylation was analyzed by immunoblotting with anti-p-Rsk (Ser386). The positions of the different myc Rsk2 constructs are indicated by black arrows to the left of the blots. The positions of molecular mass markers (in kilodaltons) are shown to the right of the blots. The results shown are representative of the results from two independent experiments.

We showed previously that p38 does not activate Rsk directly but via its downstream effector MK2/3, bypassing the requirement for the Rsk CTKD. However, the C-terminal portion of Rsk might still be needed to permit noncanonical MK2/3 phosphorylation of Rsk either because it is involved in maintaining a favorable conformation of the hydrophobic motif of Rsk and/or because it harbors a binding site, either for MK2/3 or an adaptor that brings MK2/3 together with Rsk. To determine the structural requirements for the p38-MK2/3 pathway of Rsk2 activation, we generated a series of truncation mutants some of which are shown in Fig. 2A. Progressive deletion from the C terminus first removed residues known to be required for Rsk binding to Erk1/2, including 2 critical arginine residues (22) to yield Rsk2 (1–728). We generated a further mutant lacking the C-terminal kinase domain and a substantial part of the linker region [Rsk2 (1–390)]. These Rsk mutants were myc tagged and cloned into retroviral vectors to allow expression in primary Rsk2-null DC. The cells were stimulated with LPS, and phosphorylation of Ser386 was measured along with the phosphorylation sites in the CTKD and NTKD which are targeted by Erk1/2 (S577) and PDK1 (S227), respectively. As expected, the full-length, myc-tagged Rsk2 (1–740) was phosphorylated on all these sites in response to LPS signaling (Fig. 2B). Phosphorylation of S577 was blocked as expected by PD184352, while phosphorylation of S386 and S227 was blocked only in the presence of PD184352 and BIRB0796, consistent with Erk1/2- and p38-driven activation pathways in DC (Fig. 2B). Removal of the Erk1/2 binding site [Rsk2 (1–728)] prevented phosphorylation of S577 but not phosphorylation of S386 or S227. Remarkably, phosphorylation of these two key residues was sustained in the more drastically truncated forms of Rsk2 (Fig. 2B), which lack all of the C-terminal domain and much of the linker region [Rsk2 (1–390)]. Moreover, loss of the CTKD in Rsk2 (1–390) rendered Rsk activation completely resistant to PD184352 and fully sensitive to BIRB0796 (Fig. 2B). In other words, these truncations produced a form of Rsk that is exclusively activated by the p38 pathway in DC. These results demonstrate that the C-terminal region of Rsk is dispensable for Rsk activation in DC. To assess whether the truncated forms of Rsk were catalytically active as implied by sustained phosphorylation of S386 and S227, we isolated myc-tagged Rsk2 (1–390), Rsk2 (1–728), Rsk2 (1–740) as well as endogenous Rsk2 and tested their kinase activity *in vitro*. As shown in Fig. 2C, LPS stimulated the activity of both the truncated Rsk2 products. Moreover, in contrast to full-length Rsk2, blockade of the p38 pathway substantially inhibited activation of truncated Rsk2 lacking the Erk1/2 binding domain or the C-terminal kinase domain (Fig. 2C). These results contrasted dramatically with those obtained in MEF where neither Rsk2 (1–728) or Rsk2 (1–390) could be phosphorylated by LPS (Fig. 2D).

### Distinct partitioning of p38, MK2/3, and Rsk in DC may partly explain its unusual mode of activation.

**FIG 3:**
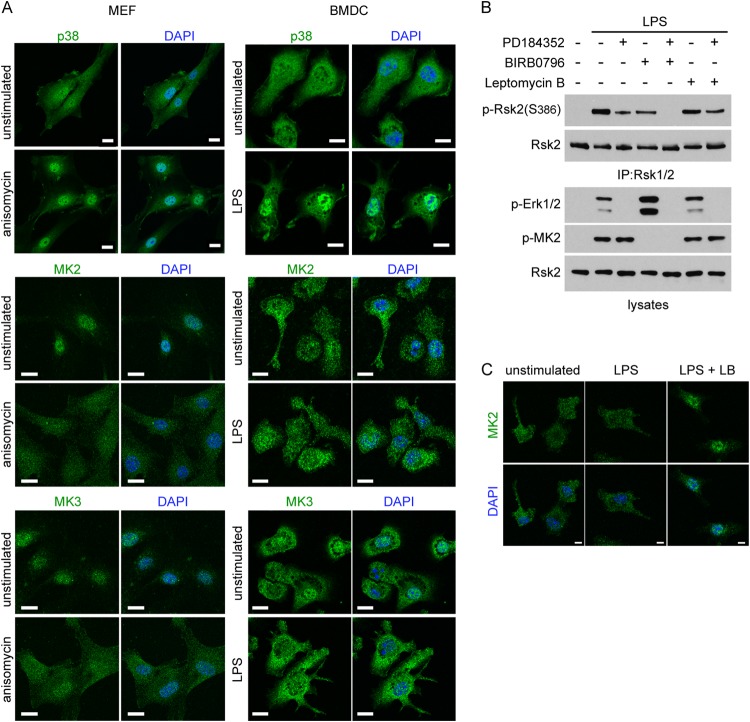
Constitutive cytoplasmic MK2/3 mediates Rsk phosphorylation. (A) MK2 and MK3 localization in MEF or BMDC left untreated or stimulated with anisomycin (10 ng/ml) or LPS (50 ng/ml) for 30 min. The cells were fixed, permeabilized, and stained with anti-p38, anti-MK2, or anti-MK3 antibodies. Fluorescence was analyzed on a LSM700 Zeiss confocal microscope. The images shown are representative of the images from three independent experiments. Bars, 20 μm. (B) BMDC were left untreated or stimulated with LPS (50 ng/ml) for 30 min in the absence or presence of 2 μM PD184352, 0.1 μM BIRB0796, or 50 nM leptomycin B. Phosphorylation of Ser386 from immunoprecipitated Rsk2 was analyzed by immunoblotting. Cell lysates were immunoblotted for p-Erk1/2, p-MK2, and total Rsk2. The blots shown are representative of the blots from three independent experiments. (C) BMDC were stimulated as in panel B either in the absence or presence of 50 nM leptomycin B (LB), and MK2 localization was analyzed by immunofluorescence. The images shown are representative of the images from three independent experiments. Bars, 20 μm.

Since most cells express p38, MK2/3, and Rsk isoforms, it is surprising that the noncanonical pathway of Rsk activation under study here is confined to DC and macrophages. One possibility is that these hematopoietic cells express an adaptor protein that brings MK2/3 and Rsk together. Although the truncation analysis reported above does not eliminate this possibility, it would limit the regions of Rsk that could engage such an adaptor to the NTKD and part of the linker region. Another possibility is that the spatial distribution of p38, MK2/3, and Rsk isoforms is distinct in DC and macrophages. In fibroblasts, MK2 and probably MK3 are located predominantly in the nucleus as a complex with p38α due to the presence of nuclear localization sequence (NLS) motifs in MK2/3 proteins (23, 24). Upon activation, p38α phosphorylates MK2, unmasking the MK2 nuclear export sequence, which results in the cytoplasmic relocalization of the p38/MK2 complex (24, 25). We compared the localization of MK2, MK3, and p38 in BMDC and MEF. In MEF, p38 was present in both the cytoplasm and nucleus, whereas MK2 and MK3 were localized in the nucleus (Fig. 3A). Upon stimulation, p38 accumulated in the nucleus, and MK2 and MK3 translocated into the cytoplasm, as previously described. Remarkably, in resting BMDC, MK2 and MK3 were not only present in the nucleus but were also found in the cytoplasm (Fig. 3A). As in MEF, the remaining nuclear pool of MK2/3 translocated into the cytoplasm upon activation of p38 by LPS.

We hypothesized that the striking constitutive cytosolic location of MK2/3 prior to activation by p38 in DC might be relevant to MK2/3-driven Rsk activation in these cells. To test this, we took advantage of the inhibitor leptomycin B, which blocks the nucleus-to-cytosol export of proteins (24), reasoning that if cytoplasmic MK2/3 were responsible for Rsk phosphorylation in BMDC, its upstream activation by p38 and downstream activation of Rsk should be resistant to leptomycin B. Indeed, as shown in Fig. 3, MK2/3 phosphorylation in response to LPS was abolished by p38 inhibition as expected but was resistant to leptomycin B (Fig. 3B). This contrasted with previous data which showed that in 293T cells a form of MK2 that lacked the NLS, and therefore was localized in the cytoplasm, could not be activated (23). Importantly, phosphorylation of S386 on Rsk2 was also resistant to leptomycin B given alone or in combination with the Erk1/2 pathway inhibitor PD184352 (Fig. 3B). Leptomycin B was active in these experiments, because leptomycin B treatment induced the accumulation of greater amounts of MK2 in the DC nucleus (Fig. 3C). However, some remained in the cytosol, and consistent with this, even prolonged leptomycin B treatment did not inhibit Rsk2 S386 phosphorylation (data not shown). Taken as a whole, these results demonstrate an atypical cytosolic localization of p38/MK2/3 in DC and show that p38-driven Rsk activation in DC did not require nuclear export of p38/MK2/3. Whether the differential localization of the upstream activators of Rsk in DC fully accounts for the noncanonical pathway will require further investigation.

### Erk1/2- and p38-activated Rsk may utilize partially distinct substrates.

**FIG 4:**
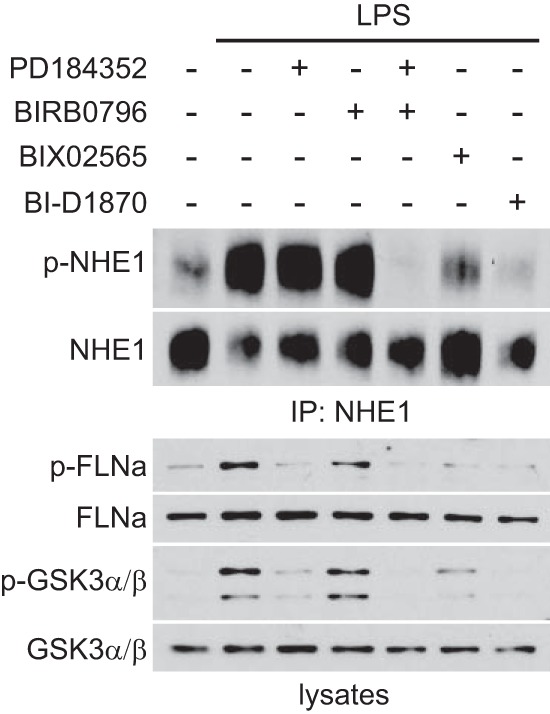
Rsk activated by the canonical and noncanonical pathways utilize partially distinct substrates. BMDC were left untreated or stimulated with LPS (50 ng/ml) for 30 min in the absence or presence of 2 μM PD184352, 0.1 μM BIRB0796, 2 μM BIX02565, or 4 μM BI-D1870. NHE1 was immunoprecipitated, and phosphorylation was analyzed by immunoblotting. Cell lysates were immunoblotted for phosphorylated filamin A (p-FLNa), total FLNa, p-GSK3α/β, and total GSK3α/β. The results shown are representative of the results from three independent experiments.

Using three well-characterized Rsk substrates, the Na^+^/H^+^ transporter NHE1 (26), the cytoskeletal protein filamin A (27), and the Ser/Thr kinase GSK3α/β (28), we asked whether the pathway of Rsk activation affected substrate preference. As shown in Fig. 4, phosphorylation of Ser707 on NHE1 was strongly enhanced by LPS signaling and almost completely blocked by the Rsk inhibitor BI-D1870 (29) or BIX02565 (30). Our recent results show that the latter compound is a more specific and reliable Rsk inhibitor (19). Rsk-dependent phosphorylation of NHE1 persisted when either the Erk1/2 pathway or p38 pathway was blocked by PD184352 or BIRB0796, respectively, and eliminated only when both pathways were inhibited (Fig. 4). Thus, NHE1 can be phosphorylated by either canonically (Erk1/2) or noncanonically (MK2/p38) activated Rsk. Interestingly, the situation was different for filamin A and GSK3. LPS-stimulated phosphorylation of filamin A on Ser2152 was reduced to background level by inclusion of PD184352 and unaffected by BIRB0796, while GSK3α/β phosphorylation on Ser21/Ser9 was predominantly blocked by PD184352 and fully blocked by the combined inhibition of Erk1/2 and p38 pathways. Thus, while NHE1 in DC appears to be fully phosphorylated via either canonical or noncanonical pathways of Rsk activation, filamin A and most GSK3 appear to be phosphorylated only by the canonical Erk1/2-dependent pathway.

### In pDC, Rsk is mainly activated by the p38 pathway and is involved in IFN-β production.

Our results thus far identify some of the structural requirements for p38-driven Rsk activation and indicate how this pathway might operate in DC but not in other cells. They also show that p38-MK2/3-activated Rsk can phosphorylate downstream substrates. However, there are additional important questions. For example, are there physiological situations that take advantage of this pathway of Rsk activation and what outputs in DC require Rsk signaling? Together, these questions imply that DC may need to respond to stimuli that preferentially activate the p38 pathway rather than the Erk1/2 pathway and that those responses require Rsk activation.

**FIG 5:**
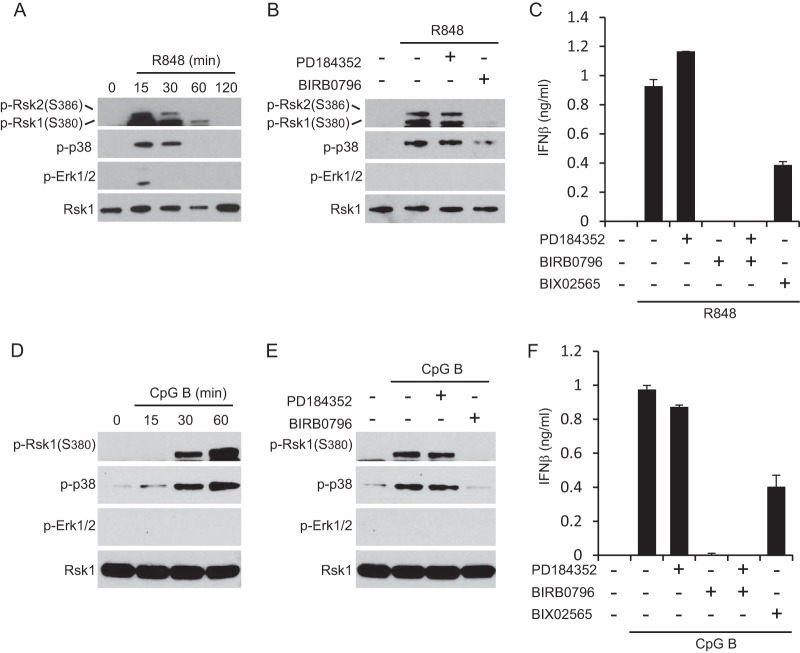
The p38 pathway activates Rsk in pDC. (A and D) pDC were stimulated with 1 μg/ml R848 (A) or with 0.1 μM CpG B (D) for the indicated lengths of time at 37°C. (B and E) pDC were left untreated or stimulated with 1 μg/ml R848 for 30 min (B) or with 0.1 μM CpG B (E) at 37°C in the presence or absence of 2 μM PD184352 or 0.1 μM BIRB0796. (A, B, D, and E) Lysates were immunoblotted with anti-pRsk (Ser386/380), p-p38, p-Erk1/2, and Rsk1 antibodies. The results shown are representative of the results from two independent experiments. (C and F) IFN-β production in pDC stimulated with 1 μg/ml R848 or 0.1 μM CpG B for 16 h at 37°C in the presence or absence of 2 μM PD184352, 0.1 μM BIRB0796, or 2 μM BIX02565. Values are the means plus SD of triplicate determinations and are representative of the data from two independent experiments.

In BMDC, activation of both Erk1/2 and p38 by TLR ligands is strong and sustained (3, 18), but these cells do not fully represent DC subset heterogeneity *in vivo*. Other culture systems generate additional DC types, including pDC which respond to viral infection by producing large amounts of type I IFN. We expanded pDC from bone marrow precursors using Flt3 ligand (31) and stimulated them with the TLR7 ligand R848. As shown in Fig. 5A, Erk1/2 phosphorylation was weak and transient, whereas p38 phosphorylation was strong and more sustained. Similar results were obtained in pDC stimulated with the TLR9 ligand, CpG (Fig. 5D). Analysis of Rsk phosphorylation showed that despite weak Erk1/2 activation, it was also strong and sustained. Moreover, blockade of the p38 pathway prevented Rsk activation in pDC (Fig. 5B and E). Thus, in pDC stimulated with TLR7 or TLR9 agonists, Rsk activation was completely p38 dependent. Next we investigated MAP kinase pathway requirements for pDC responses to TLR signaling. We stimulated pDC in the presence of MAP kinase or Rsk inhibitors and measured IFN-β production. As shown in Fig. 5C and F, production of this cytokine was unaffected by suppression of Erk1/2 signaling, consistent with weak activation of this pathway, but highly sensitive to inhibition of the p38 pathway. Importantly, IFN-β production was also substantially inhibited by the Rsk inhibitor BIX02565. Thus, p38-driven Rsk signaling is critical for optimal activation of this hallmark pDC response.

## DISCUSSION

We previously identified a novel configuration of the MAP kinase pathway in DC whereby the p38-activated kinase MK2 or MK3 activates Rsk, thus bypassing the canonical mode of Rsk activation which requires the C-terminal kinase domain (CTKD). The pathways converge at Ser386 (Rsk2) in the hydrophobic motif, which then recruits PDK1, leading to NTKD activation and downstream substrate phosphorylation. It is worth noting that Rsk CTKD and MK2/3, which, respectively, phosphorylate Ser386 in the canonical and noncanonical configuration, are closely related phylogenetically (1). These earlier results left unanswered several important questions concerning the structural basis of p38/MK2/3-driven activation of Rsk, its apparent restriction to DC, and its physiological significance. We have addressed some of these questions here.

We found that the CTKD and downstream Erk1/2 binding domain were dispensable for Rsk activation in DC and that deletion of these regions created a form of Rsk that was exclusively p38/MK2/3 activated. Whether the MK2/3 interaction with Rsk2 is facilitated by a yet unidentified scaffolding protein or is transient and unassisted is not clear. We tried to coimmunoprecipitate MK2/3 with Rsk but observed only small amounts of MK3 associated with Rsk following LPS stimulation. It appears that the interaction of Rsk2 with MK2/3 is weaker than that with Erk1/2, transient, and perhaps stimulus dependent. Among the cell types we tested, noncanonical Rsk activation was observed in DC and macrophages but not in B cells or MEF. The operation of the pathway in macrophages is consistent with the results of an earlier study (8).

Seeking a potential explanation for cell type-specific differences in Rsk activation pathways, we observed a striking difference in the distribution of MK2 and MK3 between DC and MEF. In MEF, most MK2/3 were found in the nucleus, whereas in DC, a substantial proportion was also localized to the cytosol and therefore topologically accessible to Rsk. Leptomycin B, which blocks the export of MK2/3 from the nucleus in other cell types (24), had no effect on LPS-stimulated, MK2-dependent Rsk activation in DC. MK2 and MK3 shuttle between the nucleus and cytosol, but at steady state, they reside principally in the nuclei of most cells, the NLS dominating over a nuclear export signal (NES) whose core residues are sequestered in the catalytic core until activation releases this domain (24, 32, 33). How this balance is altered in DC, favoring a constitutive cytosolic pool of MK2/3, merits further investigation. Conceivably, a binding partner exists that masks the nuclear localization sequence on MK2/3 and/or the NES is functional in the absence of upstream phosphorylation.

Although the distinct distribution of p38 and MK2/3 in DC is likely to be relevant to the operation of the noncanonical pathway, the initial experimental results suggest that simply relocating MK2/3 to the cytosol is not sufficient to establish noncanonical activation of Rsk in fibroblasts. We expressed MK2 carrying mutations that render it both constitutively active (24) and due to inactivation of the NLS, located in the cytosol of NIH 3T3 cells. However, Rsk was not activated under these conditions. One possibility is that other factors expressed only in myeloid cells are additionally needed to bridge p38/MK2/3 and Rsk.

In most cells, p38 activates three of the four MAP kinase-activated kinases, MK2/3, Msk1/2, and Mnk1/2 (1), whereas in DC, MK2/3 connects p38 to an additional fourth group of downstream kinases, the Rsks. The existence of this pathway has two implications. The first implication is that there are instances in DC and/or macrophages where Rsk signaling is required but the canonical upstream Erk1/2 pathway is only weakly activated, and the second is that there are important outputs of Rsk signaling in DC. Differential activation of p38 versus Erk1/2 MAP kinases by different TLR stimuli has been reported in DC where TLR4 or TLR5 ligands promoted stronger p38 activation and TLR2 signaling drove stronger Erk1/2 activation (34). Also, in human CD14^+^ monocytes stimulated with a TLR8 agonist, p38 activation was much stronger than Erk1/2 activation, whereas in the CD14^dim^ monocyte subset, the situation was reversed (35). We found a clear example of weak Erk1/2 activation in pDC stimulated with the TLR7 agonist R848 and TLR9 agonist CpG B. pDC are an important subset of DC that execute several immunological functions but are particularly associated with production of type I IFNs that are crucial in antiviral responses (36). In complete contrast to the situation in, for example, fibroblasts, Rsk activation in pDC downstream of TLR7 signaling was entirely driven by the p38 pathway and was minimally affected, if at all, by Erk1/2 inhibition. This important result illustrates that there is considerable plasticity in the “wiring” of the MAP kinase pathway and that paradigms established in cell lines and in other primary cells do not apply universally. We further show that p38 inhibition blocks type I IFN production in pDC stimulated with a TLR7 ligand and that Rsk activity downstream of p38 is important for this response. Relocating the principal domain of p38/MK2/3 activation from the nucleus to the cytosol increases the number of potential substrates for the p38 pathway in DC, and Rsk may not be the only pathway to benefit. For example, in a recent study, it was shown that in macrophages MK2 is implicated in the phosphorylation of Akt1/2 (37) though probably indirectly through modulation of membrane PIP3 levels. The p38/MK2/3/Rsk pathway operating in DC may have another important role in inflammatory cytokine signaling. Although Erk1/2 and p38 pathways have both been implicated in the regulation of cytokine production, several studies have shown that the intensity of Erk1/2 activation generally correlates with the amount of the anti-inflammatory cytokine interleukin-10 (IL-10) produced which in turn can have an inhibitory effect on the production of proinflammatory cytokines (38). The p38 pathway has been shown to exert negative feedback on Erk1/2 activation (39, 40), but the mechanism is not fully characterized. Because Rsk is known to yield negative feedback on Erk1/2 activity (29, 41, 42), we speculate that p38-driven activation of Rsk also serves to suppress Erk1/2 activation. In other words, the noncanonical pathway under study here may serve to further polarize MAP kinase activity away from Erk1/2 and toward p38.

Several studies, including some studies of DC (43, 44) have shown that blockade of p38 suppresses type I IFN production, but to our knowledge, our results are the first to show a link between Rsk activity and production of these key inflammatory cytokines. Further studies are needed to explore this link. mTOR (mammalian target of rapamycin) might be relevant here, since an earlier study reported that inhibition of mTOR, or some of its downstream mediators, prevented interferon regulatory factor 7 (IRF7) activation by TLR9 and consequently inhibited IFN-α/β production (45). Rsk can activate mTOR by inactivating Tsc1/2, which acts as a GTPase-activating protein (GAP) for the mTOR-activating GTPase Rheb (46).

Finally, unregulated production of type I IFNs may lead to DC and B cell activation in the context of autoimmunity, and indeed, pDC responses to self rather than pathogen-derived nucleic acids have been linked to pathologies such as systemic lupus erythematosus and psoriasis (47). A great deal of attention has been focused on suppressing inflammatory cytokines through p38 inhibition (13). Our results imply that Rsk might be considered an alternative downstream drug target in situations where type I IFNs are linked to pathology.
